# Chiral Nematic Structure of Cellulose Nanocrystal Suspensions and Films; Polarized Light and Atomic Force Microscopy

**DOI:** 10.3390/ma8115427

**Published:** 2015-11-18

**Authors:** Derek G. Gray, Xiaoyue Mu

**Affiliations:** Department of Chemistry, McGill University, Pulp and Paper Building, 3420 University Street, Montreal, QC H3A-2A7, Canada; xiaoyue.mu@mail.mcgill.ca

**Keywords:** cellulose nanocrystals, chiral nematic order, polarized light microscopy, fingerprint texture, reflection colours, coffee-stain effect, sessile droplets, contact line pinning

## Abstract

Cellulosic liquid crystalline solutions and suspensions form chiral nematic phases that show a rich variety of optical textures in the liquid crystalline state. These ordered structures may be preserved in solid films prepared by evaporation of solvent or suspending medium. Film formation from aqueous suspensions of cellulose nanocrystals (CNC) was investigated by polarized light microscopy, optical profilometry and atomic force microscopy (AFM). An attempt is made to interpret qualitatively the observed textures in terms of the orientation of the cellulose nanocrystals in the suspensions and films, and the changes in orientation caused by the evaporative process. Mass transfer within the evaporating droplet resulted in the formation of raised rings whose magnitude depended on the degree of pinning of the receding contact line. AFM of dry films at short length scales showed a radial orientation of the CNC at the free surface of the film, along with a radial height variation with a period of approximately *P*/2, ascribed to the anisotropic shrinkage of the chiral nematic structure.

## 1. Introduction

Cellulosic liquid crystalline materials, both cellulose derivatives [1,2] and cellulose nanocrystals [3,4] form chiral nematic phases that show a rich variety of optical textures in the liquid crystalline state. The ordered structures in the liquid crystalline state may be preserved with modification in solid films prepared by simple evaporation of solvent or suspending medium.

Polarized light microscopy, supplemented by scanning probe microscopy and electron microscopy provides an accessible tool to observe the structure of these ordered suspensions and films. Here, we present some observations on the chiral nematic self-organization of cellulose-based systems, with emphasis on the aqueous suspensions of cellulose nanocrystals (CNC) and of films formed from CNC by evaporation. The factors that govern phase separation, chiral nematic pitch, mechanical properties and applications of CNC and other cellulosic nanocrystals have been reviewed elsewhere, [5,6,7] and will not be discussed in detail here.

## 2. Results and Discussion

### 2.1. Fluid Cellulosic Chiral Nematic Textures

#### 2.1.1. Fluid Phases, Fingerprint Texture

**Figure 1:**
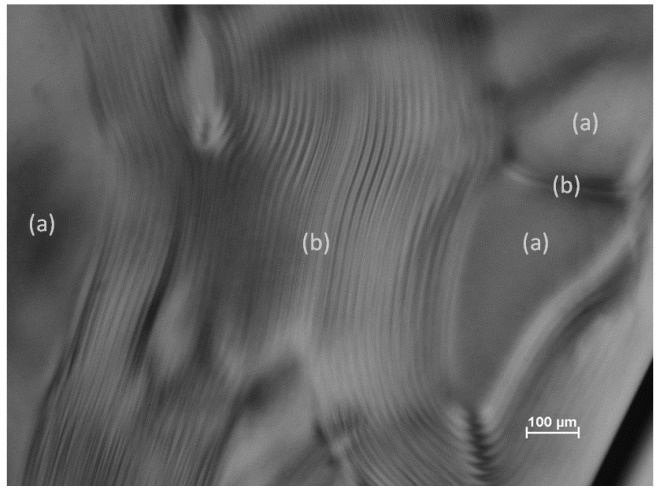
Image of 8% aqueous suspension (Batch SB-8%, see experimental section) of cellulose nanocrystals in glass microslide. Regions (a) show planar texture and (b) show fingerprint texture (see Figure 2).

Above some critical concentration, suspensions of cellulose nanocrystals (CNC) form chiral nematic phases [3]. The liquid crystalline textures observed by polarized light microscopy depend on the orientation of the optical axis of the helicoidal assembly relative to that of the microscope. Figure 1 shows a typical image of an aqueous CNC suspension with regions (a) of planar texture and (b) of fingerprint texture, where the chiral nematic axis is parallel to and orthogonal to the plane of the sample respectively.

**Figure 2:**
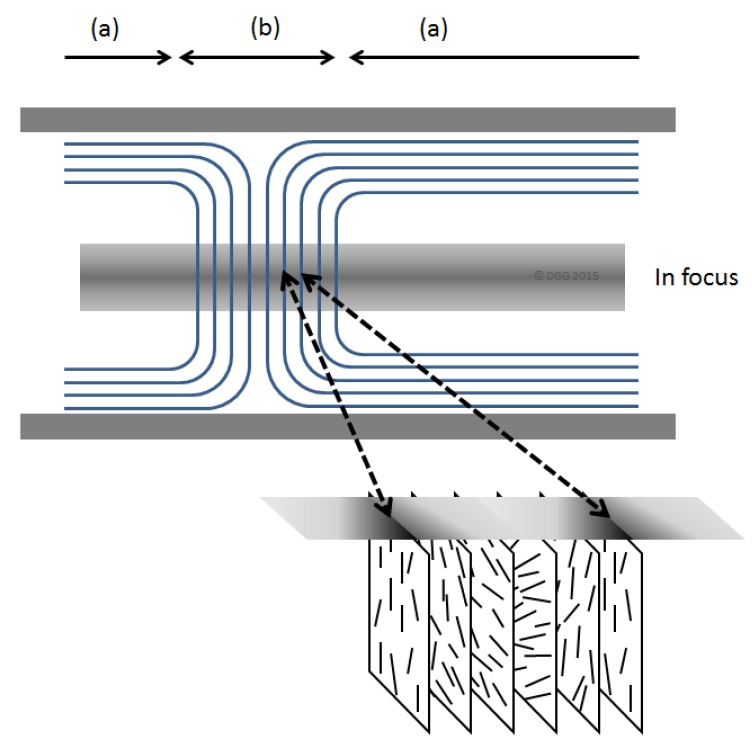
Simplified sketch of organization of chiral nematic textures shown in Figure 1. Regions (a) and (b) correspond to planar and fingerprint textures, respectively. The optical axis of the microscope is from top to bottom of the sketch, with the middle of the sample in focus. The light and dark lines under crossed polars originate from variations in birefringence due to the orientation of the cellulose nanocrystals (CNCs) along and across the microscope optical axis [8].

A variety of disclinations [8] where fingerprint lines intersect are also visible. The distance between the lines in the fingerprint texture is *P*/2, where *P* is the pitch of the chiral nematic structure. The orientation of the CNC rods is shown in the idealized sketch in Figure 2. Over time, the CNC tend to orient parallel to the walls of the microslide, so that that the chiral nematic axis becomes orthogonal to the plane of the sample, and the fingerprint texture disappears. However, by appropriate application of a magnetic field to the CNC samples, unidirectional fingerprint textures over extended areas may be generated [9].

#### 2.1.2. Flow Distortion of Fingerprint Texture

**Figure 3:**
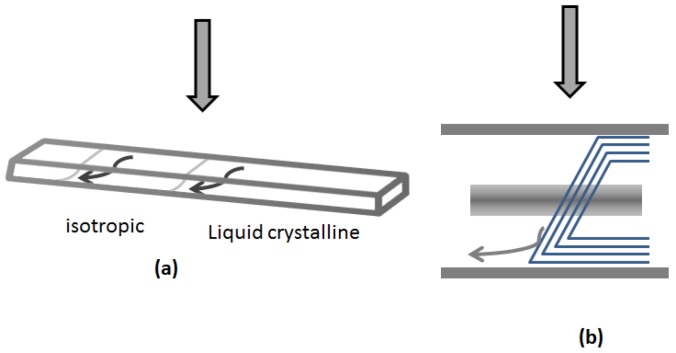
Distortion of isotropic-liquid crystalline phase boundary caused by flow of the sample fluid when placed on the microscope stage. (**a**) Biphasic sample in glass microslide; (**b**) Cross-section of phase boundary. Viewing direction indicated by arrows.

**Figure 4:**
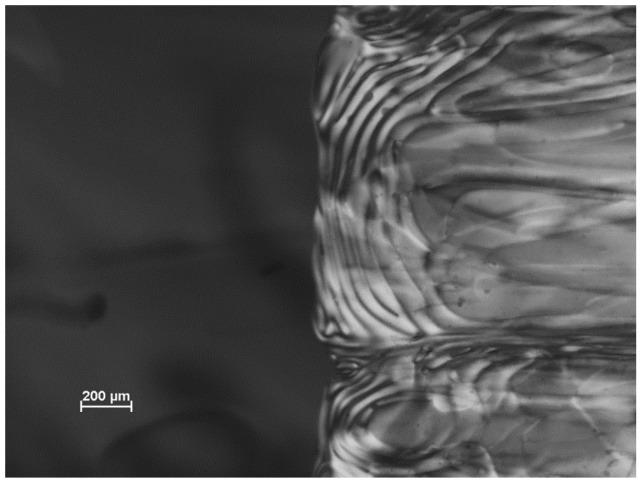
Polarized light image of the isotropic-chiral nematic phase boundary for an aqueous 6% CNC suspension (SB-6%). The isotropic phase (dark under crossed polars) is on the left, the chiral nematic phase is on the right. The sample is oriented as sketched in Figure 3.

**Figure 5:**
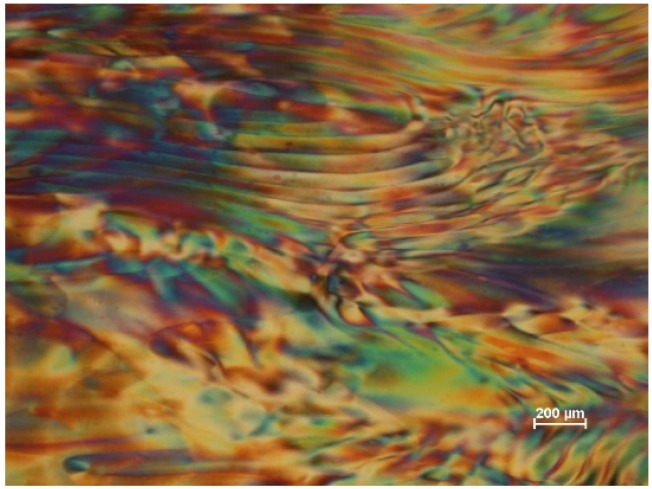
Polarized light microscope image (crossed polars, wave plate) of 6% aqueous CNC suspension (SB-6%), viewed between crossed polars with 530 nm red wave plate. The colors are due to the variations in orientation of the birefringent suspension.

As the concentration of CNC is increased, the chiral nematic phase is first observed in a biphasic region, where the isotropic and chiral nematic phases co-exist [3]. On standing, a sharp boundary forms between the upper isotropic phase and the lower liquid crystalline phase [10,11]. However, fluid samples in sealed microslides are normally rotated from the vertical position to a horizontal position for viewing with a polarized light microscope, and the resultant sample flow causes distortion of the observed texture, as sketched in Figure 3 and illustrated in Figure 4. The effect of the gravity-driven flow at the interface leads to an apparent increase in the spacing of the fingerprint lines, due to the oblique viewing angle. A similar distortion is also observed in the bulk of the liquid crystalline phase (Figure 5).

As the concentration of CNC in the suspensions increases, the chiral nematic pitch should in principle decrease to values of the order of visible light, where iridescent colours should be visible. However, before such concentrations are reached, freezing-in of the pitch due to gelation and glass formation may occur [12].

#### 2.1.3. Cellulosic Liquid Crystalline Solutions that Reflect Visible Light

**Figure 6:**
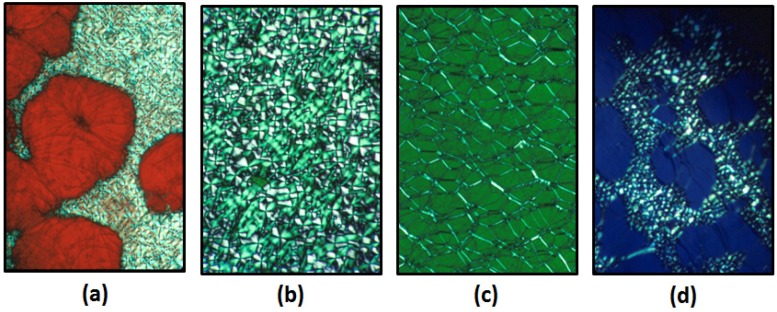
(Hydroxypropyl)cellulose (HPC)/water polarized light microscope, crossed polars, transmission images (**a**) ~45% HPC, planar and focal conic textures; (**b**) 55% HPC, focal conic texture; (**c**) 55% HPC, oily streak texture; (**d**) ~65% HPC, planar and focal conic textures. The colours of the planar regions between crossed polars correspond to the reflection colours of the samples.

While CNC suspensions don’t usually achieve pitch values small enough to reflect visible light, molecularly dispersed solutions of cellulose derivatives often show iridescent colours due to reflection of circularly polarized light of visible wavelengths, although the colours take a long time to form, presumably because the polymer chains take a long time to disentangle and adopt chiral nematic order. Thus, for the (hydroxypropyl)cellulose (HPC)-water system [1], the effect of polymer concentration on chiral nematic pitch is shown in Figure 6. These images were photographed by transmitted light between crossed polars. Thus, isotropic regions should be black, birefringent regions should be bright and planar chiral nematic regions in Figure 6a,c,d should display the colour of circularly polarized light transmitted by the sample. (The other hand of circularly polarized is reflected from the sample.) The samples appear red (Figure 6a), green (Figure 6b) and (Figure 6c) and blue (Figure 6d) to the naked eye, irrespective of the texture. The textures are characteristic of typical low molar mass chiral nematic liquid crystals, but the samples are very viscous, and some textures require time (weeks) to form.

**Figure 7:**
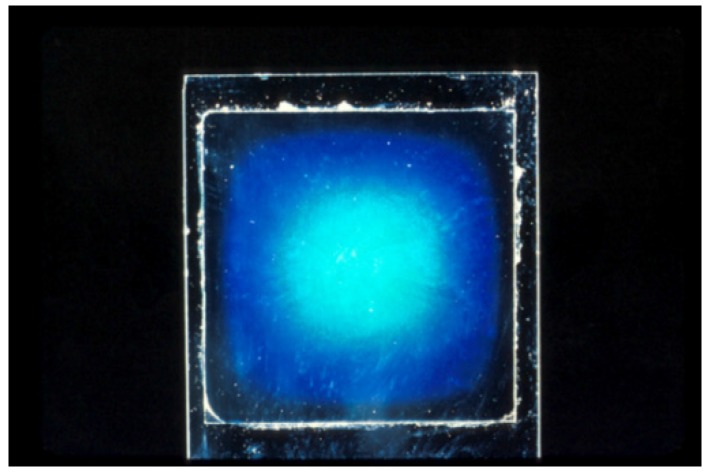
Reflection color from a thin 65 wt % solution of HPC in water, between a microscope slide and a 22 mm square cover glass. The sample is illuminated by white light against a black background. Evaporation of water leads to a higher concentration and shorter reflection wavelength at edge of sample.

An example of the reflected light from a blue HPC sample illuminated by white light against a black background is shown in Figure 7. In view of the discussion below, it should be noted that the wavelength of reflected light, and hence the chiral nematic pitch, at the edge of the sample is smaller than that at the centre of the sample. In fact, the edge of the HPC sample shown in Figure 7 is clear, suggesting that the reflection wavelength has moved into the ultraviolet region as the sample dries.

### 2.2. Solid Cellulosic Films with Chiral Nematic Properties

Cellulose and cellulose derivatives have long been exploited for their film-forming properties. Examples include cellophane, the first transparent food wrap, and cellulose acetate, whose use as base for photographic films has been replaced by its use as protective and passive optical elements in liquid crystal flat-screen displays. If these film-forming properties could be combined with their tendency to form chiral nematic solutions and suspensions, the unique optical properties of chiral nematic liquid crystals would become available in a cheap easily handled solid film, based on an essentially renewable resource. To date, the cellulose derivatives have proved difficult, in general forming viscous solutions and solids that are slow to develop chiral nematic organization. CNC suspensions in water are less viscous, and chiral nematic suspensions and films are more readily prepared. However, control of the orientation and the CNC in dry films remains challenging. In this work, we focus on CNC films produced by simple evaporation of water from a sessile droplet of CNC suspension on a flat surface. Hoeger *et al.* [13] showed by means of a convective-shear assembly that shear and capillary forces generated alignment parallel and normal to the withdrawal direction of a deposition plate, respectively. Both shear and capillary forces might be expected to act on the orientation of CNC deposited at the edge of an evaporating droplet. It is of interest to test which effect predominates.

#### 2.2.1. Evaporation of Water from Edge of Covered Chiral Nematic CNC Suspensions

**Figure 8:**
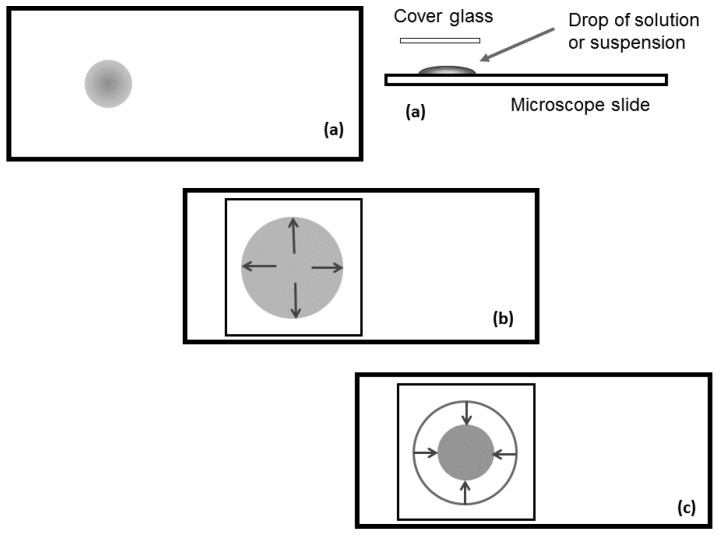
Sketch of sample preparation between microscope slide and cover glass. (**a**) Sessile droplet with finite contact angle on microscope slide; (**b**) Droplet spreads under cover glass; (**c**) Droplet shrinks due to edge evaporation.

For samples sandwiched between microscope slide and cover glass, evaporation can only occur from the edge of the sample, resulting in a concentration gradient decreasing from the edge to the centre of the sample. This gradient will influence the orientation of the nanocrystals at the point where they are frozen into the solid. Other factors will also affect the orientation and texture of the film produced on drying (Figure 8). When placing the cover glass on top of a droplet on the microscope slide (Figure 8a), the droplet is forced to spread as it forms a thin layer of uniform thickness (Figure 8b). This process generates both radial and transverse shear forces on the anisotropic and viscoelastic liquid crystalline fluid. Low viscosity samples will be drawn to the edge of the cover glass by capillary forces. If the contact lines between the fluid and the slide and coverglass are not pinned, then the droplet radius may shrink during evaporation (Figure 8c).

**Figure 9:**
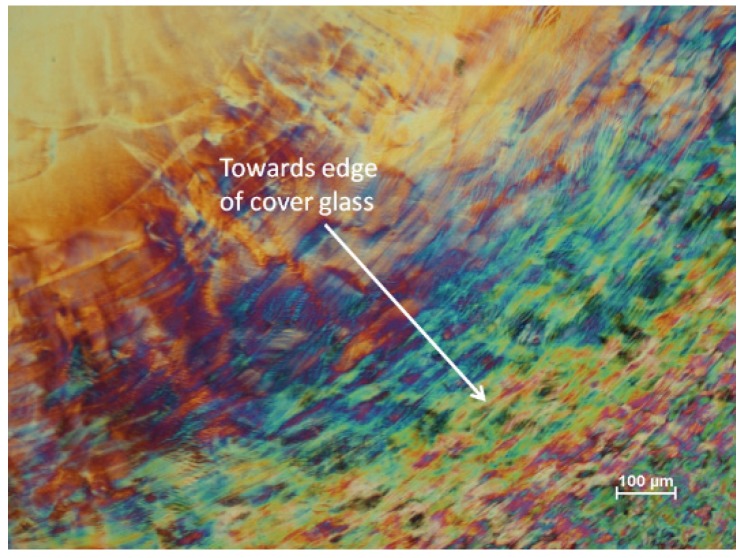
Texture (polarized light microscope, crossed polars, 530 nm red wave plate) of 8 wt % (Batch SB-8%) CNC suspension on drying between microscope slide and cover glass.

Complex textures are observed for dry films of 8% CNC suspensions allowed to dry between microscope slide and cover glass (Figure 9). For this sample and CNC concentration, the diameter of the dry film was the same as that of the initial droplet between slide and cover glass. Thus the contact lines between the drying droplet and the glass surfaces must have been pinned, and the evaporation of water from the edge of the sample resulted in a decrease in thickness only; the situation sketched in Figure 8c did not apply here. The graininess of the texture was smallest close to the edge of the sample (bottom right of the image), increasing towards the centre of the cover glass. What appear to be residual areas of distorted fingerprint texture are evident away from the sample centre. This suggests that the relatively rapid drying at the edge of the sample generates a polydomain structure, but as the process proceeds, the domains enlarge until, at the centre, the sample assumes an essentially planar texture, oriented with the chiral nematic axis orthogonal to the microscope slide and cover glass.

#### 2.2.2. Evaporation of Water from Uncovered Droplets of Chiral Nematic CNC Suspensions

**Figure 10:**
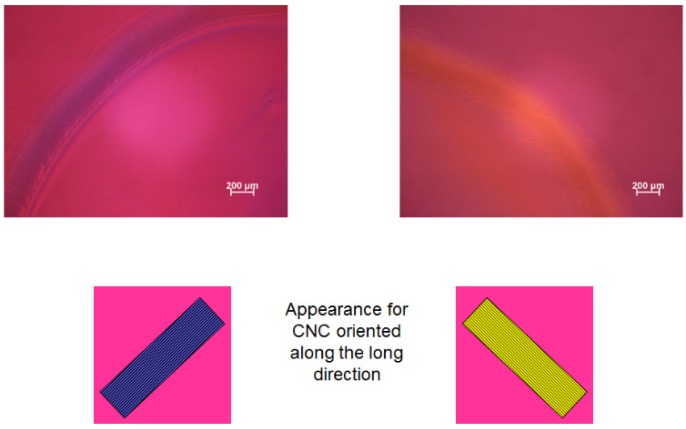
Polarized light image (crossed polars, 530 nm red wave plate) of dry film left on glass microscope slide by evaporating droplet of dilute (initially 0.52 wt %) aqueous CNC suspension. Two segments of the disc-shaped residue are shown. The blue and yellow colours observed with the 530 nm red wave plate [15] indicate that the birefringent CNC are oriented radially, as indicated in the lower sketches.

The edge-wise evaporation of water from a sample of CNC suspension between glass slide and cover glass is slow and impractical on a larger scale. What happens if the droplet is left uncovered? Aqueous droplets containing low concentrations of rod-like CNC have been reported to display a nematic-like arrangement at the edge of the ring [14]. The orientation of CNC in the residue deposited on a glass slide by the evaporation of a dilute suspension is readily observed by polarized light microscopy with 530 nm red plate (Figure 10). The 530 nm red plate generates a red colour for isotropic material and blue and yellow colours for birefringent material oriented as indicated in the sketches. The colours are faint, because of the dilute suspension left a thin film on the glass. The quadrants occupied by the blue and yellow colours indicate that the orientation direction of the nanocrystals deposited by the evaporating droplet is radial (parallel to the edge of the droplet).

Evaporation of suspensions above the critical concentration for liquid crystal formation showed a more dramatic effect. Preliminary observations of the evaporation of droplets starting from CNC concentrations high enough to form chiral nematic suspensions showed the formation of films with a remarkable 3-Dimensional iridescent pattern [16]. The contact line at the edge of the droplet was pinned, and the CNC in colloidal suspension were transported during evaporation to the outer edge of the droplet by the so-called “coffee-stain” effect. Here we examine the evaporation process in more detail, focusing on the textures observed by polarized light microscopy and on the topology of the films observed by profilometry and atomic force microscopy.

The drying process initially causes a reduction in chiral nematic pitch, if for no other reason that the concentration of chiral centres increases with increasing concentration. Thus, there are no reports of the reflection of circularly polarized light from *fluid* CNC suspensions, but iridescence is often observed for *films* made from such suspensions [17,18,19,20,21].

**Figure 11:**
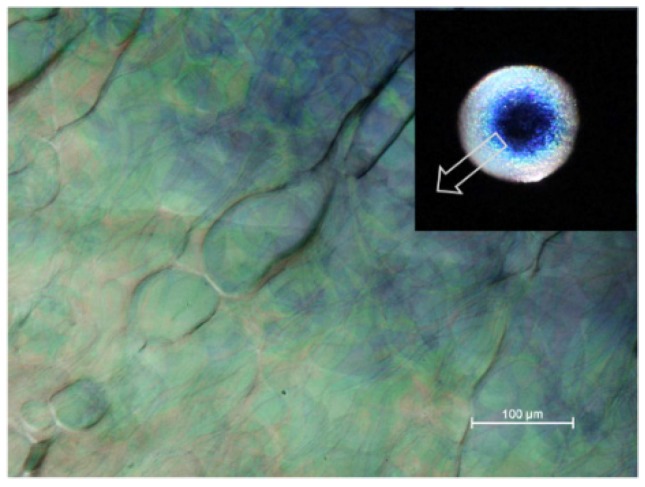
Polarized light image (transmission, between crossed polars) of film cast from 5.2 wt % (SA-5.2%) aqueous CNC suspension on glass. The insert shows a reflection image of a similar droplet dried on a black acrylic surface. The observed iridescent region is indicated by the arrow on the insert.

An example of the texture of an iridescent film is shown in Figure 11. The reflection band colours range from blue to red, as indicated on the insert, although the colours towards the edge of the droplet are less intense due to the thickening and misalignment of the sample due to the coffee-stain effect [16]. The dark lines are disclination lines at the edge of regions of essentially planar orientation. No fingerprint lines are present, because the chiral nematic pitch for the sample is in the visible region. Note that a wave plate was not used to generate the main image in Figure 11; the colours observed in transmission match those of the reflection band, as explained for the much more intense colours shown by the (hydroxypropyl)cellulose/water system (Figure 6).

The freely dried droplets show another difference from edge evaporation, in that the progression of colours from edge to centre is reversed. For covered droplets, evaporation from the edge leads to a higher concentration and hence a shorter pitch at the edge, as shown for example in Figure 7. For freely dried droplets, as mentioned above, the longer wavelength reflections are near the edge. A similar trend has been observed by Lagerwall *et al.* [4] and Dumali *et al.* [22]. However, for vacuum-dried CNC films, the colours were blue at edge and red at centre [20]; evidently the rate of drying influences the colour sequence.

**Figure 12:**
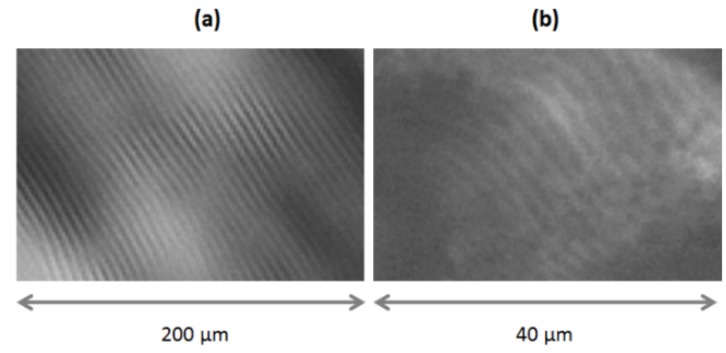
Fingerprint textures, polarized light microscope, crossed polars. (**a**) 5.2 wt % aqueous CNC suspension (Batch SA-5.2%); (**b**) Film cast from same suspension.

Even where iridescent reflection is not observed, the pitch values, indicated by fingerprint line spacings (*P*/2) are much reduced on drying (Figure 12). In solid films, the line spacing is only visible over small areas of the film, and the spacing often varies with location because the chiral nematic axis is not in the plane of the film so that the apparent line spacing is larger than *P*/2 due to the oblique viewing angle.

#### 2.2.3. Evaporation of Water from Droplets of Chiral Nematic Suspensions: Stick-Slip Textures

**Figure 13:**
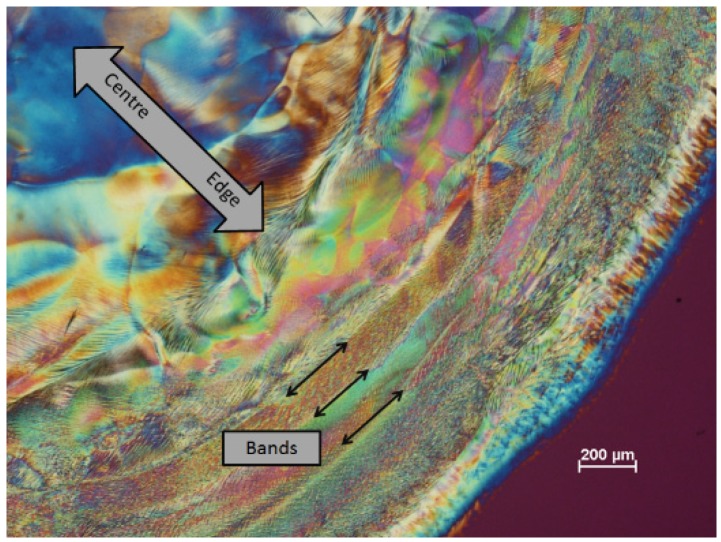
Polarized light image (crossed polars, 530 nm red wave plate) of film cast from 6 wt % CNC suspension (SB-6%) on glass.

**Figure 14:**
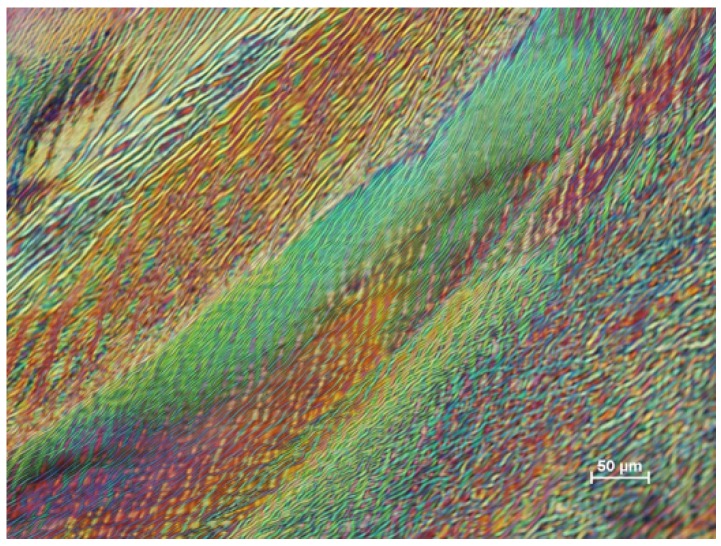
Polarized light image (crossed polars, 530 nm red wave plate) of film cast from 6 wt % CNC suspension (SB-6%) on glass showing banded texture at higher magnification.

**Figure 15:**
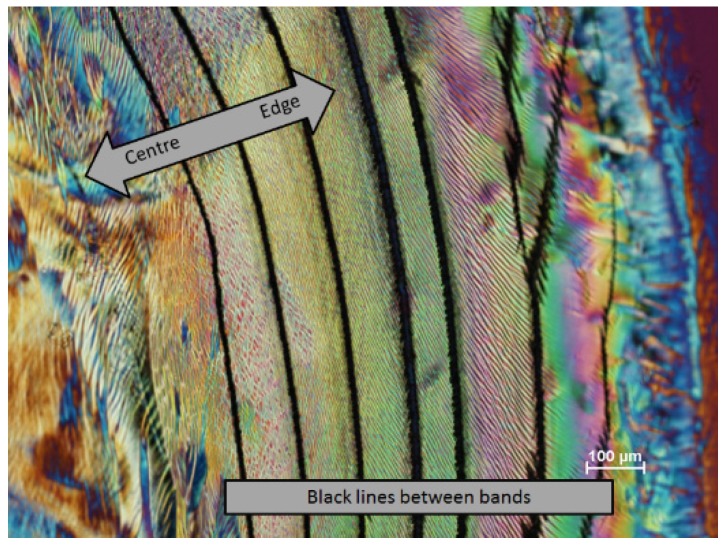
Polarized light image (crossed polars, 530 nm red wave plate) of film cast from 6 wt % CNC suspension (SB-6%) on glass showing region with cracks between bands.

With a new batch of CNC, some novel textures were observed with the polarized light microscope. A distinct banded structure was observed near the edge of the film (Figure 13). At the very edge of the film (bottom right, next to the red area indicating the isotropic glass support) a thin blue band shows that the nanocrystals in this region are oriented radially. Moving towards the centre of the sample are several distinct radial bands with more complex textures. At higher magnification, these radial bands, of the order of 100 μm wide, are themselves seen to be made up of more or less radially aligned wavy features about 2 μm wide extending for up to several hundred micron (Figure 14). Their appearance suggests a distorted fingerprint pattern. Towards the centre of the samples, the texture becomes more uniform, the orientation of the features becomes more random, and the spacing becomes larger. The radial banded structure becomes more apparent at some locations on the film, where intense dark lines appear between the rings (Figure 15). The complex nature of these images generated by the stick-slip evaporation process shows that the orientation of the nanocrystals is far from a simple chiral nematic planar or fingerprint texture.

#### 2.2.4. Profilometry of Film Topography

**Figure 16:**
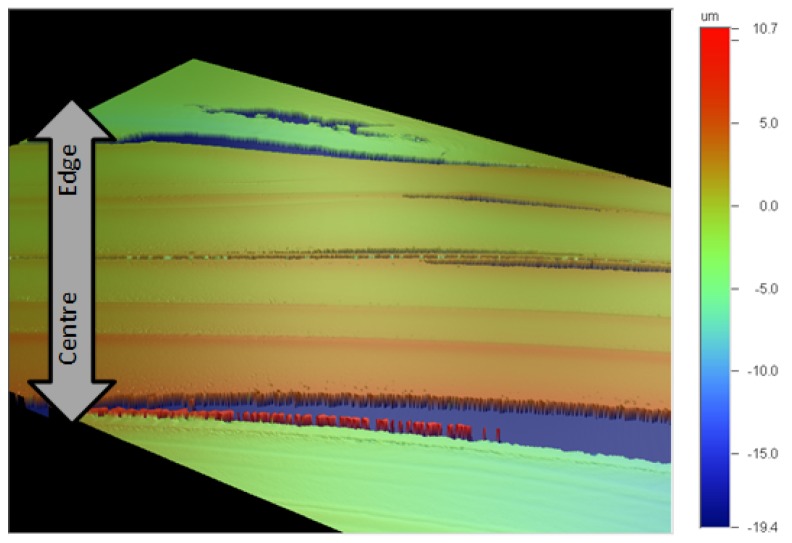
Optical profilometry of gold-coated CNC film. Perspective view showing increasing step heights towards the centre of the film. Blue regions indicate cracks in the film.

To try and interpret these images, profilometer scans and atomic force microscopy (AFM) images of the surface of the dried droplets were obtained. As a first observation by means of optical profilometry, there appear to be concentric rings of material, with raised edges. However, the rings appeared to be discontinuous, and the sample signal was very noisy. It seemed that the oriented and birefringent nature of the CNC film was interfering with the optical interferometric measurements used by the instrument. To avoid this problem, samples were coated with gold to provide an optically homogeneous surface, and the samples were scanned with the optical profilometer (Figure 16). In this case a clearer topography emerges, with rings of increasing thickness towards the centre of the film. The edges of the rings are often raised, and clearly the films contain radial cracks (coloured blue) that show the level of the underlying glass surface.

**Figure 17:**
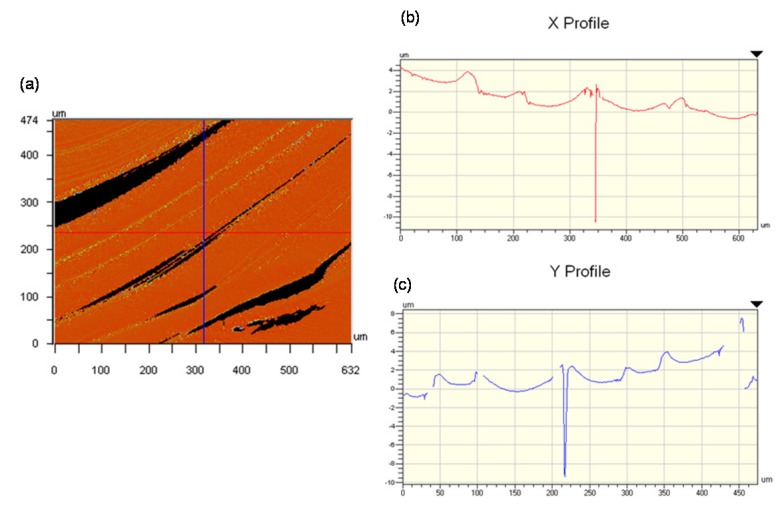
Optical profilometry of gold-coated CNC film. (**a**) Topographic image, showing ridges (yellow) and cracks (black); (**b**) Corresponding X profile; (**c**) Y profile across image.

**Figure 18:**
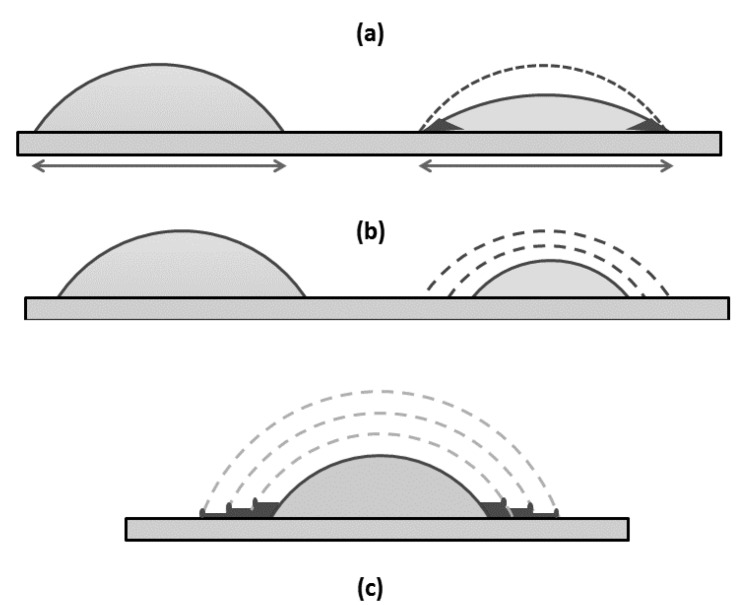
Sketch of droplet evaporation under (**a**) pinned and (**b**) stick-slip conditions; (**c**) Multiple coffee-stain deposition stages are shown, with rings of increasing thickness and raised edges.

The heights of steps and cracks are shown in the X and Y profiles across the film (Figure 17) are of the order of 2 μm, which is much smaller than the >50 μm height difference previously observed at the edge of droplets with pinned contact lines [16]. However, the profilometry shows that the outer edge of each step is also raised, suggesting that a minor “coffee-stain effect” is also occurring during evaporation. Thus a multiple “stick-slip” situation is occurring in this case, as sketched in Figure 18. The radial nature of the cracks observed in Figure 15, Figure 16 and Figure 17 indicates that there exists an anisotropy in mechanical properties of the dry films that must reflect the local orientation of the CNC. As with any fibrous solid, the strong direction of films would be expected to be aligned along the direction of the rod-like CNC, with the weakest direction normal to the CNC orientation. The radial alignment of the CNC thus favors radial cracking during drying.

**Figure 19:**
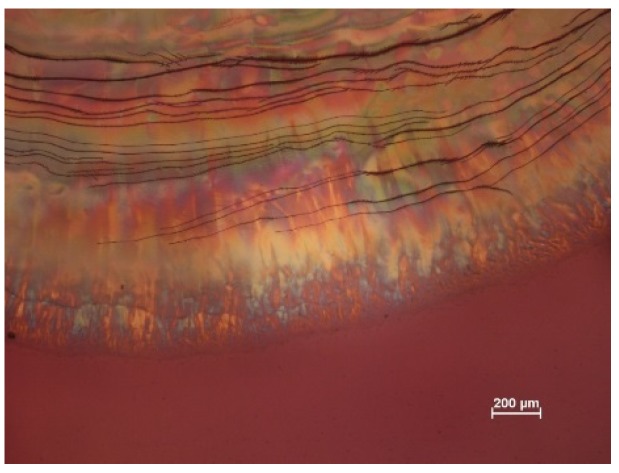
Polarized light microscope (crossed polars, 530 nm wave plate) of film cast from 8 wt % CNC suspension (SB-8%) containing 2 wt % added propane-1,2-glycol (PG).

The contrast between the relatively high ring of CNC deposited at the edge of the evaporating droplet for a pinned contact line [16] and the much more diffuse effect noted above suggests that the tendency for contact line pinning is controlling the texture. Accordingly, we tried to decrease the pinning tendency by modifying the suspension with a suitable additive. Small amounts of a water-soluble compound, propane-1,2-diol (propylene glycol, PG), were added to the evaporating droplet. PG has a lower surface tension and vapour pressure than water, and so should become enriched near the contact line during evaporation. The presence of the PG rich fluid at the contact line inhibits contact line pinning during evaporation. Figure 19 shows a polarized light image of the film prepared with PG; it shows none of the banding shown by PG-free suspensions (Figure 16, Figure 17 and Figure 18), and while dark lines attributed to cracking do occur, they are much closer together than in the case films prepared from PG-free suspensions. Thus the presence of the PG, presumably as a layer on the surface of the glass, decreases the tendency for contact line pinning. Recently, dramatic effects on contact line pinning and droplet mobility have been observed for water-PG droplets on high-energy glass surfaces [23]. While the glass surfaces that we used had not been subjected to the rigorous procedure used by Cira *et al.*, nevertheless addition of low volatility low surface tension components such as PG to CNC suspensions would be expected to alter contact line pinning and hence the orientational order of the resulting film.

### 2.3. Atomic Force Microscopy of CNC Orientation and Topography in Dry Films

**Figure 20:**
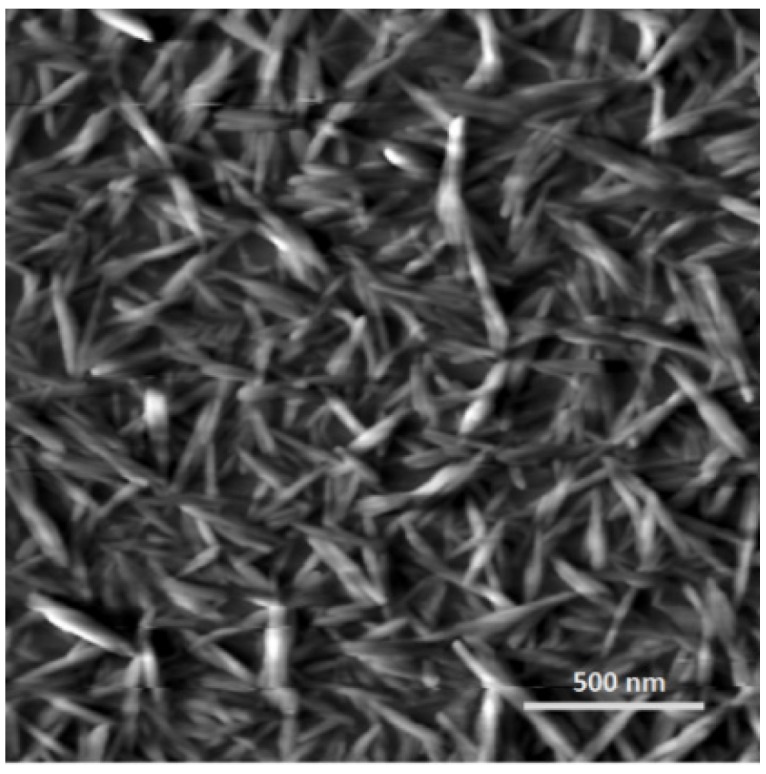
Atomic force microscope (AFM) height image of surface of film cast from droplet of isotropic (0.0032 wt %) CNC suspension.

The profilometric measurements reported above demonstrate the large scale deviations from planarity generated by colloidal forces during evaporation from anisotropic suspensions of CNC. At much higher magnifications, the orientations of individual nanocrystals in solid films may be observed. For films cast from dilute CNC suspensions, below the critical concentration for liquid crystal formation, the orientation of the nanocrystals is essential random (Figure 20), despite the fact that the suspension must pass through the concentration range where chiral nematic phase is expected. The ordering phenomenon is presumably slower than evaporation, or alternatively gelation occurs below the critical concentration for liquid crystal formation for this sample.

**Figure 21:**
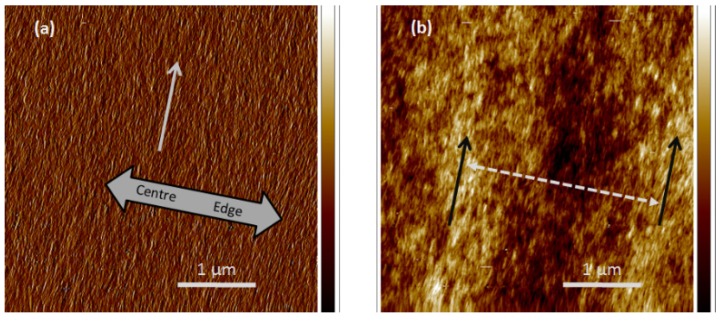
AFM (**a**) amplitude and (**b**) height images of an area of film cast from droplet of 8 wt % aqueous CNC suspension (SB-8%) on a mica substrate. The white arrow (a) indicates the approximate orientation of the nanocrystals; the black arrows (b) indicate the approximate orientation and spacing of ridged areas of the sample. The height colour scale in (b) is from −19 to +19 nm.

The surface orientation of CNC in films cast from suspensions above the critical concentration for chiral nematic phase formation is quite different. Atomic force microscope (AFM) images of a typical area of a film cast from an 8 wt % CNC suspension on a mica substrate are shown in Figure 21. The amplitude mode image (a) shows that the CNC are fairly uniformly oriented parallel to the edge of the sample. The height mode image (b) shows that the surface is uneven, with ridges of the order of 10 nm high and around 2–3 μm apart, also oriented parallel to the edge of the sample. A similar periodic relief at the surface of a chiral nematic oligomer has been observed by AFM, and interpreted as relating to the half-helical pitch [24]. Out-of-plane AFM deformations that correspond to optical observations on dry CNC films have been observed for parabolic focal conic and fingerprint textures [25]. The spacing of the ridges observed by AFM is of the same order of magnitude as the line spacings of the fingerprint pattern observed by optical microscopy. It is reasonable to assume that the anisotropic orientation of the CNC in the film is the cause.

**Figure 22:**
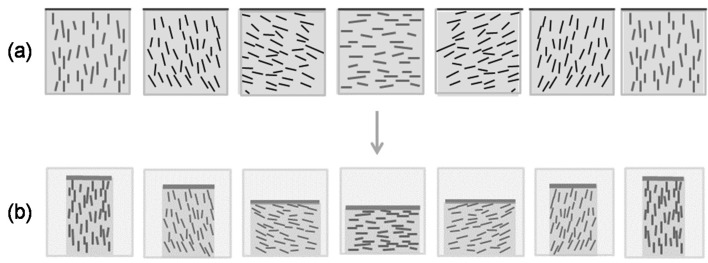
(**a**) The sketches show the orientation of CNC in sequential sections of a chiral nematic helicoid with axis normal to the plane of the page. (In other words, the orientations shown side by side should be envisaged as being one behind the other, with the sketch on the left being at the front.) The CNC concentration is assumed high enough for gel formation, and the free surface is at the top of the sketches. On evaporation (**b**) the tendency of the CNC gel to shrink to a greater extent parallel to the CNC orientation may lead to a surface ripple with a periodicity of *P*/2 along the chiral nematic axis.

A possible explanation is sketched in Figure 22. The orientation of CNC in successive adjacent nematic-like layers of a chiral nematic structure close to an open surface is shown in the upper series of cross-sections. The chiral nematic axis is taken to be perpendicular to the page, and the sketches cover one half of the chiral nematic pitch. Assume that the concentration of CNC is sufficient to form a gel. The gel properties will depend on the orientation of the CNC; in particular, gels will shrink more in directions perpendicular to the CNC and less in directions parallel to the CNC. Thus the reduction in volume will be anisotropic, resulting in a periodicity of *P*/2 in height along the helicoidal axis, where *P* is the pitch in the final dry film. This type of anisotropic swelling and shrinkage is well-known on a larger length scale; the orientation of the cellulose microfibrils along the secondary wall of wood pulp fibres causes them to shrink on drying much more laterally than longitudinally, with important consequences for the bonding and properties of paper sheet [26].

This model is clearly oversimplified; the drying process will generate in-plane as well as out-of-plane shrinkage and the chiral nematic axis may well be at an angle to the evaporating surface. However, where clear fingerprint patterns are detected optically near a free surface, a corresponding surface topology may be expected. The effect resembles the theoretical predictions of nanoscale undulations and disclination lines for appropriately anchored chiral nematic liquid crystals, driven by director surface gradients and elastic constants [27]. However, the surface distortion for CNC films is driven by the anisotropic shrinkage of a chiral nematic gel.

## 3. Experimental Section

### 3.1. CNC Preparation and Purification

All the CNC suspensions were prepared as described previously [28]. Briefly, 40 g of dried Whatman cotton powder was reacted with a preheated sulfuric acid solution (64% *w*/*w*, 700 mL) at 45 °C for 45 min, after which the reaction was quenched by 5 L of deionized water (18.2 MΩ·cm, Millipore Milli-Q Purification System, EMD Millipore, Billerica, MA, USA) and left to settle for 2 h. The suspension was repeatedly concentrated and washed by centrifugation (6000 rpm, 10 min cycles) until the supernatant appeared turbid, the suspension was then extensively dialysized against deionized water until the effluent remained at neutral pH (Spectra/Por 4, Molecular weight cut-off 12,000–14,000 Daltons). Following the removal of excess acid, the suspensions were dispersed by sonication using a Vibracel sonicator (five 7-min cycles at 60% energy output, Vibracel Sonics and Materials, Newtown, CT, USA). An ice bath was used to prevent overheating.

Two batches of CNC, labelled SA and SB were prepared. The main difference was in the ion exchange treatment used to remove excess ion content from the suspensions. Batch SA was stirred over Dowex Marathon MR-3 (Dow Chemical Co., Midland, MI, USA) for 24 h. Batch SB was also stirred over Dowex Marathon MR-3 for 24 h, but then the mixed bed resin was filtered off and replaced by Dowex Marathon C, a strong acid cation exchange resin that had previously been rinsed repeated with ethanol to remove colour. The second resin treatment was to ensure that the resin was in acid form. The suspensions were next filtered through glass microfibre filters (0.45 μm pore size) to remove any particulate impurities introduced by the sonicater tip, and concentrated by means of a Labconco RapidVap vacuum evaporation system. The concentration of the suspensions was determined gravimetrically by drying small volumes in an oven at 105 °C. Sample SA was used as a 5.2 wt % suspension, and sample SB was concentrated to give 6 wt % (SB-6%) and 8 wt % (SB-8%) suspensions.

The CNC samples were stored at −4 °C for at least 2 weeks until the suspensions had separated into isotropic and anisotropic phases. Small amounts of each sample were sealed in glass micro-slides (VitroCom, Mountain Lakes, NJ, USA) and kept standing vertically for 2–3 days before imaging by polarized light microscopy.

### 3.2. Polarized Light Microscopy

Photographs of the liquid crystalline suspensions and the dried films prepared from CNC were taken with a Nikon Eclipse LV100POL polarized light microscope and DS-Fi1 camera (Nikon, Tokyo, Japan). A 530 nm red wave plate was inserted between crossed polars to indicate CNC orientation [15]. Some images of chiral nematic cellulose derivatives were prepared as described in the cited references.

### 3.3. Fabrication of CNC Films

CNC suspensions were drop cast onto glass (for transmitted light microscopy and optical profilometry), black acrylic (for reflected light imaging) or mica surfaces (for atomic force microscopy). The suspensions were allowed to evaporate undisturbed at ambient conditions (room temperature 20–25 °C, relative humidity 20%–60%) for 12 h to give disc-shaped solid films on the glass, acrylic or mica surfaces.

### 3.4. Optical Profilometry

The topography of mm-scale areas of the dry CNC films was investigated by optical profilometry. CNC films deposited on glass substrates were scanned with an optical profilometer (Wyko NT8000, Veeco Instruments Inc., Plainview, NY, USA). The initial results were noisy, but the quality of the topographic images was much improved by coating the surface of the CNC film with a 20 nm thick layer of gold by means of a NexDep Ebeam Evaporator (Angstrom Engineering Inc., Kitchener, ON, Canada).

### 3.5. Atomic Force Microscopy

The topography of the CNC film surfaces at much smaller length scales was investigated by AFM. To prepare very dilute CNC samples on mica for AFM imaging of individual nanocrystals, a drop of poly-L-lysine (0.01 wt % in water) was placed onto a freshly cleaved mica surface (Electron Microscope Supplies, Hatfield, PA, USA), rinsed with water, followed by a drop of dilute CNC suspension (~0.005 wt %). The samples were left to dry in Petri dishes under ambient conditions. For images of CNC orientation in films cast from chiral nematic suspensions, a droplet of 8 wt % suspension was allowed to evaporate on the mica surface. A Multi-Mode-3 AFM (Digital Instruments, Bruker, Billerica, MA, USA) was used in tapping mode to image the samples, with probes obtained from NanoScience (Si, N-type, 0.01–0.025 Ω/cm, 200–400 kHz resonance frequency, with nominal tip radius of 10 nm).

## 4. Conclusions

Simple evaporation of a droplet of aqueous CNC suspension leads to a surprising range of self-organizing phenomena:

1. Starting with a dilute suspension of CNC, well below the critical concentration for liquid crystal formation, the CNC tend to align radially at the edge of the droplet.
2. For more concentrated suspensions that are initially above the critical concentration, some radial initial orientation of CNCs at the edge of the film is observed. This is in line with recent observations of the orientation of CNCs in shear flow, where the acid-form CNCs self-oriented across the flow direction [29].
3. Drying droplets of concentrated CNC display a topography governed by evaporation-driven mass transfer (the “coffee-stain effect”).
4. The magnitude of the coffee-stain effect is greatest for strongly pinned droplets, where the height near the edge of the dry film is several times the height in the middle of the film [16].
5. When pinning of the contact line is weaker, a “stick-slip” situation is observed, with layers of increasing thickness are laid down by the receding contact line. The layers give a complex optical texture; radial cracking of the drying film was sometimes observed.
6. The surface topography of dry films at short length scales showed a radial orientation of the CNC at the free surface of the film, along with a radial height variation with a period of approximately *P*/2, ascribed to the anisotropic shrinkage of the chiral nematic structure.
7. The rich liquid crystalline textures displayed by the evaporation of water from CNC suspensions depend on the interplay of concentration gradients that influence chiral nematic pitch, director orientation and gelation with the effects of surface orientation and the elastic constants normally associated with liquid crystalline phases. The qualitative results here are very preliminary, but hopefully indicate some of the variables involved in the formation of chiral nematic solids from cellulose nanocrystal suspensions.

## References

[1] Werbowyj R.S., Gray D.G. (1976). Liquid crystalline structure in aqueous hydroxypropyl cellulose solutions. Mol. Cryst. Liq. Cryst. Lett..

[2] Nishio Y., Sato J., Sugimura K. (2015). Liquid crystals of cellulosics: Fascinating ordered structures for the design of functional material systems. Adv. Polym. Sci..

[3] Revol J.F., Bradford H., Giasson J., Marchessault R.H., Gray D.G. (1992). Helicoidal self-ordering of cellulose microfibrils in aqueous suspension. Int. J. Biol. Macromol..

[4] Lagerwall J.P.F., Schütz C., Salajkova M., Noh J.H., Park J.H., Scalia G., Bergström L. Cellulose nanocrystal-based materials: From Liquid Crystal Self-Assembly and Glass Formation to Multifunctional Thin Films. http://www.nature.com/am/journal/v6/n1/abs/am201369a.html.

[5] Habibi Y., Lucia L.A., Rojas O.J. (2010). Cellulose nanocrystals: Chemistry, self-assembly and applications. Chem. Rev..

[6] Klemm D., Kramer F., Moritz S., Lindström T., Ankerfors M., Gray D., Dorris A. (2011). Nanocelluloses: A new family of nature-based materials. Angew. Chem. Int. Ed..

[7] Moon R.J., Martini A., Nairn J., Simonsen J., Youngblood J. (2011). Cellulose nanomaterials review: Structure, properties and nanocomposites. Chem. Soc. Rev..

[8] Chandrasekhar S. (1992). Liquid Crystals.

[9] Revol J.F., Godbout L., Dong X.M., Gray D.G., Chanzy H., Maret G. (1994). Chiral nematic suspensions of cellulose crystallites; phase separation and magnetic field orientation. Liq. Cryst..

[10] Dong X.M., Kimura T., Revol J.F., Gray D.G. (1996). Effects of ionic strength on the phase separation of suspensions of cellulose crystallites. Langmuir.

[11] Chen W., Gray D.G. (2002). Interfacial tension between isotropic and anisotropic phases of a suspension of rod-like particles. Langmuir.

[12] Mu X., Gray D.G. (2014). Formation of chiral nematic films from cellulose nanocrystal suspensions is a two-stage process. Langmuir.

[13] Hoeger I., Rojas O.J., Efimenko K., Velev O.D., Kelley S.S. (2011). Ultrathin film coatings of aligned cellulose nanocrystals from a convective-shear assembly system and their surface mechanical properties. Soft Matter.

[14] Uetani K., Yano H. (2013). Self-organizing capacity of nanocelluloses via droplet evaporation. Soft Matter.

[15] Introduction to Polarized Light Microscopy. https://www.microscopyu.com/articles/polarized/polarizedintro.html.

[16] Mu X., Gray D.G. (2015). Droplets of cellulose nanocrystal suspensions on drying give iridescent 3-D “coffee-stain” rings. Cellulose.

[17] Revol J.-F., Godbout L., Gray D.G. (1998). Solid films of cellulose with chiral nematic order and optically variable properties. J. Pulp Paper Sci..

[18] Beck S., Bouchard J., Berry R. (2011). Controlling the reflection wavelength of iridescent solid films of nanocrystalline cellulose. Biomacromolecules.

[19] Beck S., Bouchard J., Chauve G., Berry R. (2013). Controlled production of patterns in iridescent solid films. Cellulose.

[20] Tang H., Guo B., Jiang H., Xue L., Li B., Cao X., Zhang Q., Li P. (2013). Fabrication and characterization of nanocrystalline cellulose films prepared under vacuum conditions. Cellulose.

[21] Dumanli A.G., van der Kooij H.M., Kamita G., Reisner E., Baumberg J.J., Steiner U., Vignolini S. (2014). Digital color in cellulose nanocrystal films. ACS Appl. Mater. Interfaces.

[22] Dumanli A.G., Kamita G., Landman J., van der Kooij H.M., Glover B.J., Baumberg J.J., Steiner U., Vignolini S. (2014). Controlled, bio-inspired self-assembly of cellulose-based chiral reflectors. Adv. Opt. Mater..

[23] Cira N.J., Benusiglio A., Prakash M. (2015). Vapour-mediated sensing and motility in two-component droplets. Nature.

[24] Meister R., Dumoulin H., Halle M.A., Pieranski P. (1996). The anchoring of a cholesteric liquid crystal at the free surface. J. Phys. II.

[25] Roman M., Gray D.G. (2005). Parabolic focal conics in self-assembled solid films of cellulose nanocrystals. Langmuir.

[26] Page D.H., Tydeman P.A., Bolam F.M. (1965). Consolidation of the Paper Web.

[27] Rofouie P., Pasini D., Rey A.D. (2015). Nano-scale surface wrinkling in chiral liquid crystals and plant-based plywoods. Soft Matter.

[28] Kloser E., Gray D.G. (2010). Surface PEG-grafting of cellulose nanocrystals in aqueous media. Langmuir.

[29] Tatsumi M., Teramoto Y., Nishio Y. (2015). Different orientation patterns of cellulose nanocrystal films prepared from aqueous suspensions by shearing under evaporation. Cellulose.

